# A Public Health initiative on hepatitis E virus epidemiology, safety and control in Portugal – study protocol

**DOI:** 10.1186/s12879-016-1341-5

**Published:** 2016-01-16

**Authors:** João R. Mesquita, Mette Myrmel, Kathrine Stene-Johansen, Joakim Øverbø, Maria S. J. Nascimento

**Affiliations:** 1 Escola Superior Agrária de Viseu, Instituto Politécnico de Viseu, Viseu, Portugal; 2 Department of Food Safety and Infection Biology, Norwegian University of Life Sciences, Oslo, Norway; 3 Department of Virology, Norwegian Institute of Public Health, Oslo, Norway; 4 Departamento de Ciências Biológicas, Laboratório de Microbiologia, Faculdade de Farmácia da Universidade do Porto, Rua de Jorge Viterbo Ferreira n.° 228, 4050-313 Porto, Portugal

## Abstract

**Background:**

The discovery of autochthonous hepatitis E in industrialized countries has changed the understanding of hepatitis E virus (HEV) infection in these regions, now known to be mainly due to zoonotic transmission of genotype 3. The foodborne route of transmission via consumption of contaminated meat from HEV infected pigs is well documented as well as the direct occupational exposure to animal reservoirs. Accumulating evidence also points to an emerging potential threat to blood safety after the identification of viremic blood donors and the documentation of HEV-contaminated blood or blood products. Moreover, the origin of several iatrogenic cases remains unclear and porcine-derived pharmaceutic products have been suspected as a cause. Severe morbidity following HEV infection in patients receiving immunosuppressive therapy and in those with severe immunodeficiency from other causes has been recently recognized as a serious consequence of this infection in industrialized countries. In Portugal no large-scale HEV seroprevalence study has been undertaken, no professional risk groups have been identified, and the risk of blood donation from HEV silent infected donors is unknown. The present paper describes seroepidemiological and molecular approaches to answer these questions.

**Methods/design:**

To address these issues a study protocol was designed that will approach: i) the seroprevalence of HEV among the Portuguese general population; ii) HEV infection among butchers and slaughterhouse workers (occupational risk); iii) the silent HEV infection in Portuguese blood donors (HEV transfusion-associated risk); iv) the potential HEV contamination of porcine-derived pharmaceutical products. Commercial enzyme immunoassays and real-time/conventional RT-PCR assays will be used.

**Discussion:**

This study is the first evaluation of the seroepidemiological status to HEV infection of the Portuguese population, the first to potentially identify professional risk groups, and to evaluate the safety of blood and blood products and porcine-derived pharmaceutics in Portugal. It will generate valuable data applicable for preventive and control measures against HEV infection (e.g., introduction of systematic screening of blood donors, control of blood products or porcine derived pharmaceutical products), thus helping to manage the burden of this viral disease.

## Background

Hepatitis E virus (HEV) is a non-enveloped, positive-sense, single-stranded RNA virus of the genus *Hepevirus*, family *Hepeviridae*, with at least four recognized genotypes infecting humans, [1–4]. Genotypes 1 and 2 are mainly transmitted through the fecal-oral route due to fecal contamination of water supplies or food products and are prevalent in developing countries in Asia, Africa, and Central America, where hepatitis E is highly endemic. HEV infections in industrialized countries have been, until recently, considered only in travelers returning from HEV endemic regions. However, the discovery of autochthonous HEV in industrialized countries has changed the understanding of HEV infection in these regions, now known to be mainly due to genotype 3 and the result of zoonotic transmission [2, 3]. Swine, wild boar and deer have been described as reservoirs for the zoonotic HEV genotype 3 strains, highlighting the foodborne route of HEV transmission [5–9] and animal contact through occupational transmission showing a higher likelihood of infection in veterinarians and farmers working with HEV reservoirs [10].

Accumulating evidence indicating that HEV is endemic in industrialized countries, parallel to increasing reports of chronic hepatitis E in immunosuppressed patients and the recognition of hepatitis E extra-hepatic manifestations [11–13] needs to be further elucidated in a Public Health perspective. Hepatitis E is recognized as an emerging potential threat to blood safety in industrialized countries after the documentation of several cases of transmission by HEV-contaminated blood, blood products, or by transplantations [14]. The silent HEV infection of blood donors is today well documented. HEV RNA has been frequently found in asymptomatic blood donors in Europe with studies reporting the occurrence of HEV RNA in 1 in 8000 Swedish plasma donors [15], 1 in 4500 German plasma donors [15], 1 in 2848 in the United Kingdom [16] and 1 in 2600 Dutch blood donors corresponding to donation of HEV-positive blood once per day in the Netherlands [17]. Moreover, in several iatrogenic cases the source of HEV infection remains uncertain and porcine-derived pharmaceutical products have been suspected [18].

In Europe the prevalence of HEV immunoglobulin (Ig)G, a marker of previous exposure to HEV, in the general population varies widely, from 1.9 % [19] to 52.5 % [20]. This marked difference has been attributed to the different sensitivity and/or specificity of the anti-HEV IgG assays used [4, 21], as well as the different populations studied. In Portugal there are only a few geographically focused studies on HEV in humans, many of which were performed before the recognition of genotype 3 [22–27]. These studies have shown substantial variation in the HEV prevalence (2.1–29 %), that can not only be attributed to the different sensitivity/specificity of the anti-HEV IgG assays used, but more to the region and population studied, that included both healthy blood donors and patients with chronic viral hepatitis.

In Portugal, no large-scale HEV seroprevalence study has been undertaken, no professional risk groups have been identified, the risk of blood donation from donors with silent HEV infection and the possibility of HEV contamination of porcine-derived pharmaceuticals is unknown. The project HEPeCONTROL: HEPATITIS E VIRUS EPIDEMIOLOGY, SAFETY AND CONTROL, funded by the Public Health Initiatives Program of the European Economic Area Grants (EEA Grants; project number 60DT2), aims to fill in these knowledge gaps and will allow characterization the seroepidemiological pattern of HEV contributing to evidence based data for implementation of control measures in Public Health by:

i) providing detailed information on the prevalence of HEV infection among the general Portuguese population through the study of IgG and IgM anti-HEV in a large-scale epidemiological survey (N ~ 1600) on a representative cohort of the Portuguese population. Risk factors for HEV infection will be determined by studying the likelihood of seropositivity in relation to demographic characteristics such as sex, age and place of residence
ii) evaluating the risk of HEV infection in butchers and slaughterhouse workers (occupational risk) by studying serum IgG/IgM anti-HEV in a cohort of these professionals (N ~ 100)
iii) evaluating silent HEV infection in Portuguese blood donors (N ~ 1500) (HEV transfusion-associated risk) by screening sera for the presence of IgM anti-HEV and HEV RNA
iv) searching for potential HEV contamination of a variety of porcine-derived pharmaceutical products.

## Methods/design

### Ethics approval

This study was approved by the Portuguese ethical commission (Comissão de Ética para a Saúde do Centro Hospitalar de São João; Reference number: 99/2015). Accordingly, all participants will be informed of the aims and methods of this study and will be asked to provide their informed consent. Information and approval documents will be filled in duplicate and signed by both the participant and medical staff who will draw blood. One copy will be given to the participant and the other kept by the staff.

### Study of the prevalence of HEV infection among the general Portuguese population

In order to estimate the prevalence of HEV infection in the Portuguese population with considerable precision the sample size was calculated according to previously described methods [28]. A sample size of 1656 was calculated based upon the following assump tions: i) an *a priori* seroprevalence of anti-HEV of 50 % (yielding the highest possible sample size) [29]; ii) a confidence in the estimate of 95 %; iii) a maximum allowable error in the prevalence of 3 %; iv) a population size of Portugal of 10,541,840 [ 30 ] . A stratified random sampling design with the Nomenclature of Territorial Units for Statistics (NUTS) level III as a stratification level (a total of 30 NUTS III regions of Portugal; Table 1) was set up to provide a representative sample. On the basis of the Portuguese census data [30], the stratified distribution of the Portuguese population by NUTS level III, by sex and 5 years age group was used for setting up the sampling frame. The sampling frame will comprise of attendees of Clinical Analysis Laboratories from which blood samples will be collected until the required number of samples is reached. Blood samples will be collected and stored at −20 °C within 24 h.

**Table 1:** Sampling design according to the Nomenclature of Territorial Units for Statistics (NUTS) level III of Portugal

NUTS I	NUTS II	NUTS III
Continental Portugal	Norte	Minho-Lima
		Cávado
		Ave
		Grande Porto
		Tâmega
		Entre Douro e Vouga
		Douro
		Alto Trás-os-Montes
	Centro	Baixo Vouga
		Baixo Mondego
		Pinhal Litoral
		Pinhal Interior Norte
		Dão-Lafões
		Pinhal Interior Sul
		Serra da Estrela
		Beira Interior Norte
		Beira Interior Sul
		Cova da Beira
		Oeste
		Médio Tejo
	Lisboa	Grande Lisboa
		Península de Setúbal
	Alentejo	Alentejo Litoral
		Alto Alentejo
		Alentejo Central
		Baixo Alentejo
		Lezíria do Tejo
	Algarve	Algarve
Azores Archipelago	Azores Archipelago	Azores Archipelago
Madeira Archipelago	Madeira Archipelago	Madeira Archipelago

In order to identify putative risk factors (age, sex and place of residence) for seropositivity, univariate and multivariate logistic regression analysis will be performed. Multivariate models are most informative given that they produce adjusted odds ratios (aOR) that simultaneously measure the strength of associations between the multiple risk factors and presence of HEV serum antibodies. Crude odds ratio (cOR) and aOR will be calculated to assess the presence of confounding factors. Variables will be considered independent when the calculated cOR and aOR are similar. A likelihood ratio test will also be performed to evaluate statistical significance of risk factors with more than 2 levels, given its independence from the variables reference level. All analyses will be performed using Epicalc package in the R software (R 2.1.2.0) (R Development Core Team, 2010).

All samples will be screened for the presence of anti-HEV IgG and IgM using the enzyme immunoassays recomWell (Mikrogen GmbH, Neuried, Germany) according to the manufacturer’s instructions. These immunoassays use highly purified recombinant HEV antigens from open reading frame (ORF) 2 of genotypes 1 and 3. Both assays are considered to have good performance in terms of negative predictive value, sensitivity and specificity [21, 31]. The sensitivity is 96.3 and 96.8 % and the specificity is 98.2 and 100 % for the IgG and IgM tests, respectively (manufacturers information). Serum samples will be considered positive following the manufacturer’s stipulated cut-off and extinction values will be converted to the corresponding antibody concentration in units per ml (U/ml) using the formula (extinction sample / extinction cutoff) x 20, according to the manufacturer’s instructions.

Positive or equivocal results in the recomWell HEV IgG/IgM immunoassays will be further tested by the immunodot recomLine HEV IgG/IgM (Mikrogen GmbH, Neuried, Germany). The immunodot assay has a sensitivity of 100 % and specificity of 98.8 % (for IgG), and sensitivity of 100 % and specificity of 100 % (for IgM), according to the manufacturers information. This immunodot assay uses highly purified recombinant HEV antigens O2N (genotype 1, genotype 3), O2C (genotype 1, genotype 3), O2M (genotype 1), O3 (genotype 1, genotype 3), fixed to nitrocellulose membrane test strips. The immunodot assay and the analysis of the test strips will be performed according to the manufacturer’s instructions.

### Evaluation of HEV infection among butchers and slaughterhouse workers (occupational risk)

A case–control study will be set up with sera from butchers, slaughterhouse workers (*N* = 100) and controls (*N* = 200) from the general population matched by age, sex and region. Sera will be tested for anti-HEV IgG and IgM using the same assays as described above. The validity of the control group will be tested by a Chi-square test for unequal odds with Yates’ continuity correction.

### Evaluation of silent HEV infections in Portuguese blood donors (HEV transfusion-associated risk)

Sera from blood donors (*N* = 1500) will be screened for anti-HEV IgM (recomWell HEV IgM) and further tested by immunodot (recomLine HEV IgM) as described above. All the sera will also be screened for the presence of HEV RNA by a real-time RT-PCR [32]. Positive samples will be sequenced based on a nested RT-PCR [33] of a more variable region in ORF 1 of 330 nt. Phylogenetic analysis will be performed using MEGA 6 software to characterize the circulating strains.

### Evaluation of potential HEV contamination of porcine-derived pharmaceutical products

Five batches of 5 selected porcine-derived pharmaceutical products routinely used in hospitals or frequently sold in community pharmacies will be tested for the presence of HEV RNA by a real-time RT-PCR [32]. If positive samples are found they will be tested by nested RT-PCR [33] for genotype characterization by sequencing. Phylogenetic analysis will be performed to characterize and genotype the circulating strains.

## Discussion

The present study will provide unique insight in the potential exposure of the Portuguese general population to HEV and will generate information on risk profiles regarding demographic data (sex, age and geographical details). Further, the occupational risk of butchers and slaughterhouse workers and the risk of blood donation from HEV silent infected donors will be elucidated. Finally, the potential contamination of porcine-derived pharmaceutical products by HEV will be assessed.

Portugal has been a pioneer in the introduction of vaccines in medical practice throughout the time, being one of the best examples in Europe warranting the Portuguese population the most important vaccines for their protection. Through the 2nd National Serological Inquiry (2° Inquérito Serológico Nacional – Portugal Continental 2001–2002) the immunological profile of the Portuguese population to several diseases was collected and many important recommendations have been shared to stakeholders and decision makers in the health sector. Unfortunately, at that time HEV was not considered emergent and therefore not included in the serological inquiry. The HEPeCONTROL study aims to fill in this gap by identifying and characterizing risk factors of individuals with increased risk for exposure to HEV infection as evidenced based data for HEV vaccine recommendations in the future.

Information about the project and its results will be disseminated to both Public Health (National and International Conferences) and academic stakeholders (publication in peer-reviewed journals). The project will generate evidence based data for health authorities in Portugal for the application and implementation of preventive control measures against HEV infection (e.g., introduction of systematic screening of blood donors, control of blood products or porcine derived pharmaceutical products), thus reducing the burden of this viral disease.
